# Solitary metastasis to a superior mediastinal lymph node after distal gastrectomy for gastric cancer: a case report

**DOI:** 10.1186/s12885-018-4555-7

**Published:** 2018-06-04

**Authors:** Naoki Kubo, Junichi Yoshizawa, Takaomi Hanaoka

**Affiliations:** Department of Surgery, North Alps Medical Center Azumi Hospital, 3207-1, Ikeda, Ikeda-cho, Kitaazumi-gun, Nagano, 399-8695 Japan

**Keywords:** Gastric cancer, Mediastinum, Lymph node metastasis, Gastrectomy, Adenocarcinoma

## Abstract

**Background:**

Mediastinal lymph node metastases occasionally occur in patients of advanced gastric cancer of the cardia with esophageal invasion, but they rarely occur in patients with gastric cancer of other sites. This report describes a case of a solitary metastasis to t a superior mediastinal lymph node after distal gastrectomy for gastric cancer of the antrum.

**Case presentation:**

A 70-year-old man underwent curative distal gastrectomy for advanced gastric cancer of the antrum (pT2pN2M0, stage IIB). Postoperatively, he underwent adjuvant chemotherapy with S-1 (100 mg/day). Although the serum levels of his tumor markers increased after surgery, computed tomography scans did not detect evidence of early recurrence in the superior mediastinum. However, a ^18^F-fluorodeoxyglucose positron emission tomography (FDG-PET) scan showed accumulation of fluorodeoxyglucose in the upper mediastinum with no evidence of recurrence elsewhere. Therefore, a solitary superior mediastinal lymph node was suspected to have a metastatic lesion derived from the gastric cancer. The patient underwent tumor resection right mini-thoracotomy two years and three months following gastrectomy. A pathological examination demonstrated moderately differentiated adenocarcinoma, confirming that it was a metastatic adenocarcinoma from the gastric cancer. The patient developed recurrences in the superior mediastinum and several right costa six months following the second surgery. He was treated with chemotherapy, but he died 18 months after the second operation.

**Conclusion:**

We present a rare case of a solitary metastasis to a superior mediastinal lymph node after distal gastrectomy for gastric cancer. An FDG-PET scan is useful for the diagnosis of mediastinal lymph node metastasis in gastric cancer. Metastasis to the superior mediastinal lymph nodes from gastric cancer in sites other than the cardia suggests systemic expansion of gastric cancer, and therefore, even a solitary metastasis may be related to a poor prognosis.

## Background

Mediastinal lymph node metastases in advanced gastric cancer of the cardia with esophageal invasion occur occasionally, but metastases from sites other than the cardia are rare. Furthermore, upper mediastinal lymph node metastases from gastric cancer are often accompanied by multiple metastases to other sites (e.g., Virchow’s lymph node); therefore, cases in which a single mediastinal metastasis of gastric cancer is resected are very rare. We report a case in which a solitary metastasis to a superior mediastinal lymph node occurred after distal gastrectomy for gastric cancer of the antrum.

## Case presentation

**Fig. 1:**
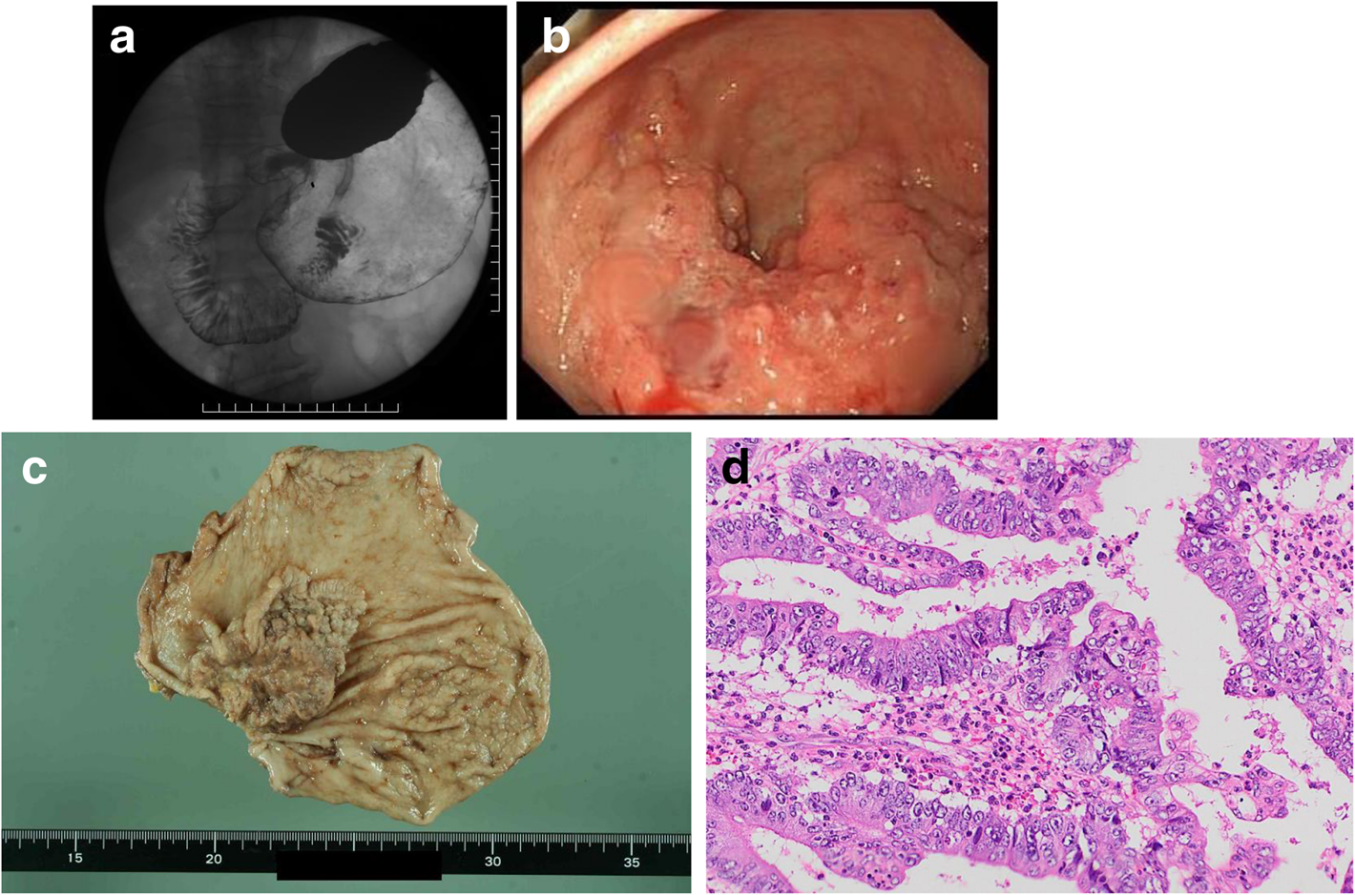
**a, b** A barium meal examination and upper gastrointestinal endoscopy revealed type III advanced gastric cancer in the antrum. **c** Resected specimens demonstrate a flat, elevated type5 advanced gastric cancer, 6.0 cm in diameter, located in the greater curvature of the antrum. **d** Histological findings of the primary tumor show moderately differentiated adenocarcinoma (hematoxylin and eosin staining, magnification, × 400)

A 70-year-old man with anemia was admitted to our hospital. A barium meal examination and upper gastrointestinal endoscopy revealed type III advanced gastric cancer in the antrum (Fig. 1a. b). Biopsy specimens from the tumor demonstrated a moderately differentiated adenocarcinoma. Laboratory examinations revealed a high level of serum tumor markers, including carbohydrate antigen (CA) 19–9 (578.5 U/mL). A computed tomography (CT) scan showed regional lymph node metastases; however, distant metastases and direct invasion to the surrounding tissues were not observed. The patient underwent curative distal gastrectomy with D2 lymphadenectomy. Resected specimens demonstrated a flat, elevated, type 5 advanced gastric. tumor that was 6.0 cm in diameter, located in the greater curvature of the antrum. The proximal margin of the resected specimen was free of residual cancer cells (85 mm) (Fig. 1c). The pathological findings of the resected primary gastric carcinoma, expressed according to the Japanese Classification of Gastric Carcinoma, were moderately differentiated adenocarcinoma, mp, INFb, intermediate, ly1, v0. Additionally, 5 of the 29 resected regional lymph nodes were positive in only the No. 6 (subpyloric) region according to the Japanese Classification of Gastric Carcinoma (Fig. 1d). The pathological stage was classified as IIB based on the American Joint Committee on Cancer TNM staging classification for carcinoma of the stomach (7th edition, 2012). The patient’ postoperative course was uneventful; his high preoperative CA19–9 level normalized (26.3 U/ml), and he was discharged.

**Fig. 2:**
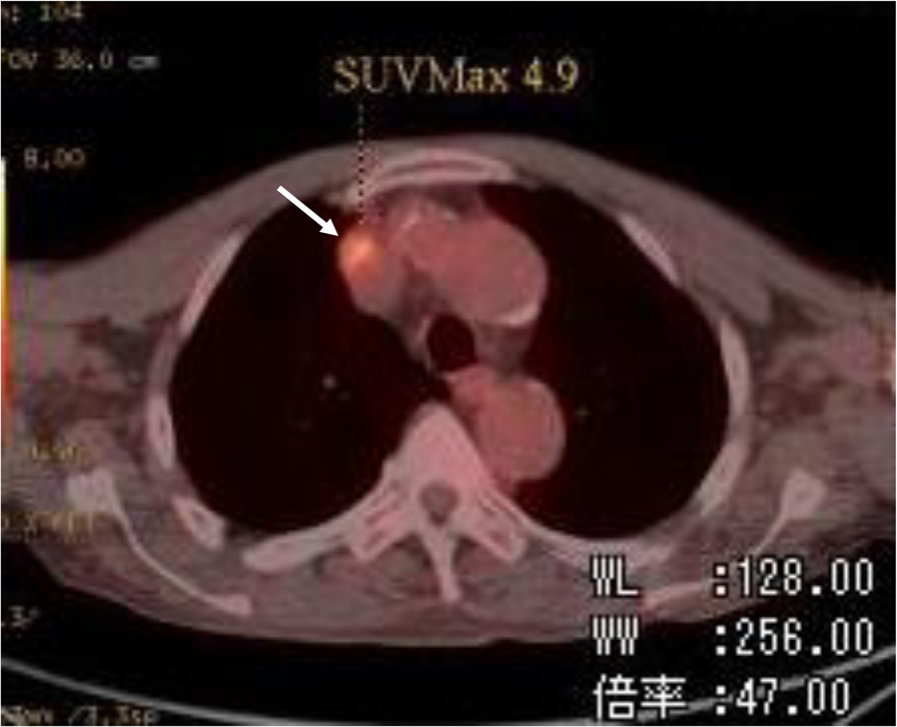
^18^ F-fluorodeoxyglucose positron emission tomography shows accumulation of fluorodeoxyglucose in the superior mediastinum but no evidence of recurrence except for that in the mediastinal lymph node *(arrow)*

**Fig. 3:**
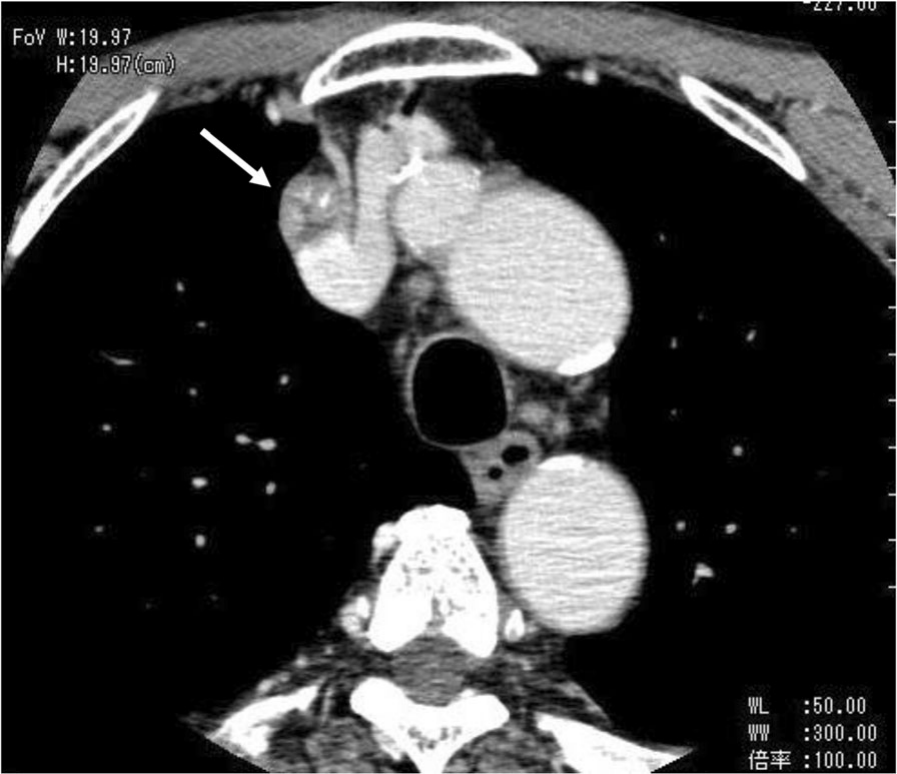
Computed tomography shows enlargement of the solitary superior mediastinal lymph node, and invasion to the right brachiocephalic vein was suspected *(arrow)*

Postoperatively, the patient underwent adjuvant chemotherapy with S-1 (100 mg/day). However, his carcinoembryonic antigen (CEA) levels ranged from 5 to 6 U/mL, and his CA 19–9 levels ranged from 40 to 120 U/mL beginning at six months after surgery. We monitored the patient via CT scans every 6 months and observed no evidence of recurrence. His tumor markers remained in that same range for several months, and therefore adjuvant chemotherapy with S-1 was continued. However, two years and two months after surgery, his CEA (12.7 U/mL) and CA 19–9 (714.0 U/mL) levels increased dramatically, and an ^18^F- fluorodeoxyglucose positron emission tomography (FDG-PET) scan was performed, which revealed an accumulation of FDG in the upper mediastinum but no other evidence of recurrence (Fig. 2). Based on these results, a repeat CT scan was performed, which revealed an enlargement of a solitary superior mediastinal lymph node (Fig. 3). The enlarged lymph node was suspected to be a metastatic lesion derived from the gastric cancer.

**Fig. 4:**
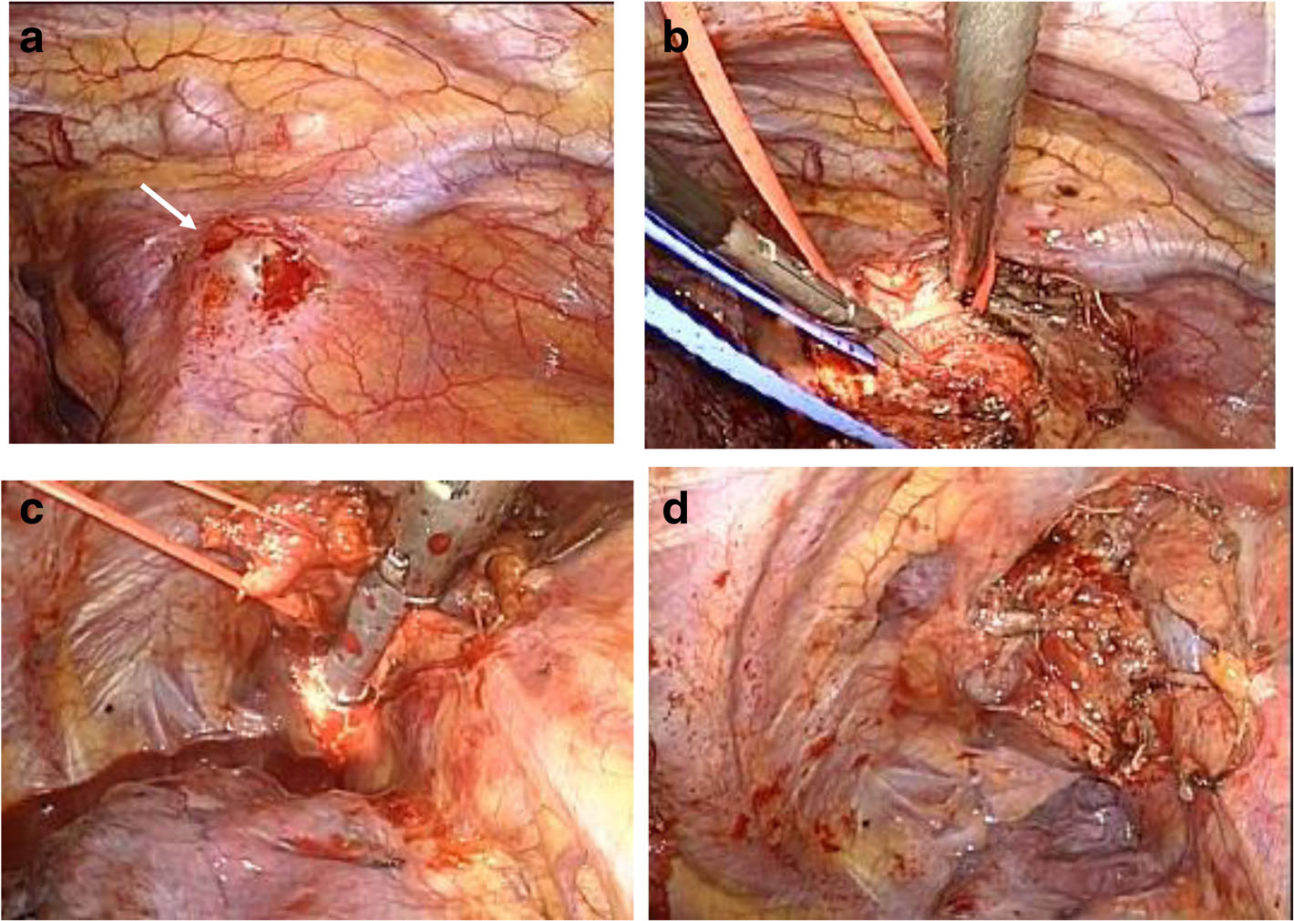
Intraoperative photography. **a** The metastasized lymph node was located in the upper mediastinum and was in contact with the right brachiocephalic vein *(arrow).* **b, c, d** The metastasized lymph node showed strong adhesion to the right brachiocephalic vein, but it could be excised with the right brachiocephalic vein

**Fig. 5:**
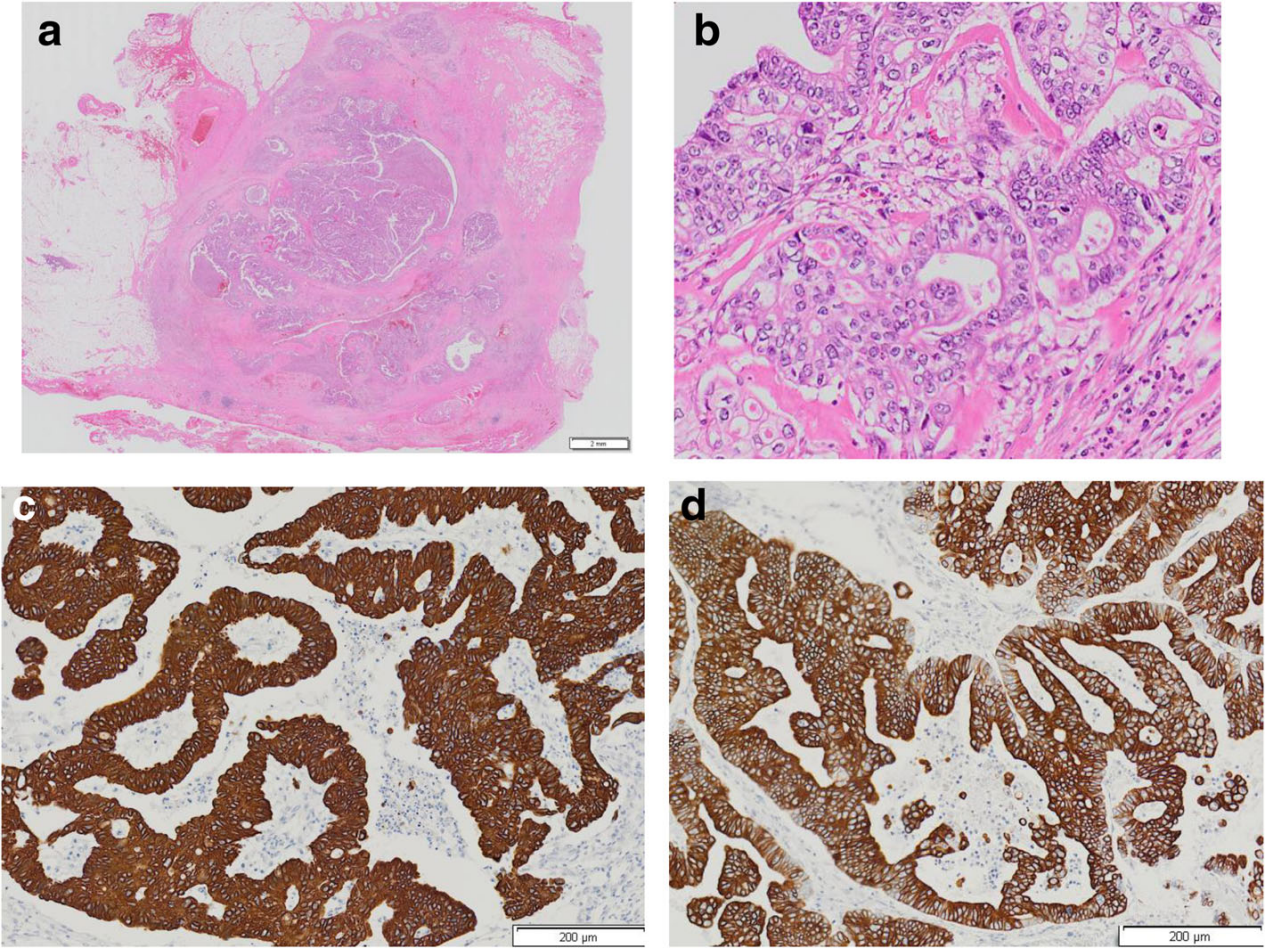
**a** The resected specimen was 1.5 cm in diameter. **b** Histological findings of the metastatic mediastinal lymph node demonstrate moderately differentiated adenocarcinoma, indicating that it was a metastasis of gastric cancer (hematoxylin and eosin staining, magnification, × 400). **c, d** Both the primary tumor and the mediastinal node exhibited partially positive immunohistochemical staining for CK7 (data not shown) and positive immunohistochemical staining for CK20 (c: primary tumor, d: mediastinal node, magnification, × 5)

The patient underwent tumor resection by right mini-thoracotomy two years and three months following the initial gastrectomy. The metastasized lymph node exhibited strong adhesion to the right brachiocephalic vein; however, it was on the periphery of the superior vena cava and therefore could be excised with the right brachiocephalic vein (Fig. 4). The patient’s postoperative course was uneventful, and he was discharged on postoperative day 17. The resected specimen was 1.5 cm in diameter (Fig. 5a), and histological examination demonstrated a moderately differentiated adenocarcinoma (Fig. 5b). Both the primary tumor and the mediastinal node exhibited partially positive immunohistochemical staining for CK7, positive immunohistochemical staining for CK20 (Fig. 5c, d), and negative staining for Her2, indicating that it was a metastatic adenocarcinoma from the gastric cancer. While the patient received adjuvant chemotherapy with S-1 (100 mg/day) following the initial surgery and because he developed recurrence, he subsequently received adjuvant chemotherapy with docetaxel (40 mg/m^2^ on days 1, 8 and 15) in a 28-day cycle after the second operation. Unfortunately, he developed recurrences in the superior mediastinum and some right costa at six months after reoperation. Therefore, he received combination chemotherapy with irinotecan (60 mg/m^2^) and cisplatin (40 mg/m^2^) every two weeks; although he had not previously received this regimen, he developed multiple mediastinal and bone metastases and died 18 months after the second operation.

## Discussion and conclusions

This case revealed two important clinical issues. First, this is a rare case of solitary metastasis to a superior mediastinal lymph node after distal gastrectomy for gastric cancer. Second, an FDG-PET scan was useful for the diagnosis of mediastinal lymph node metastasis of gastric cancer. Thus, even when solitary, the presence of superior mediastinal gastric cnacer metastases, except those from gastric cancer of the esophagogastric junction, may imply systemic expansion of gastric cancer and indicate poor prognosis.

Gastric cancer cases with esophageal invasions and gastroesophageal junction adenocarcinomas are associated with high rates of mediastinal metastasis, ranging from 16.8 to 18.1% [1, 2]. However, upper mediastinal metastasis in gastric cancer, regardless of the presence of esophageal invasion, are rare [3, 4]. Thus, superior mediastinal metastasis in gastric cancer, except for those occurring in the cardia, are rare. To our knowledge, only two documented case of superior mediastinal metastases of gastric cancer after distal gastrectomy have been reported in the Medline and Japana Centra Revuo Medicina databases; both are in patients who received chemotherapy but not surgery for superior mediastinal metastasis and multiple organ metastasis [5, 6].

Metastatic pathways include lymphangitic spread of the tumor that reaches the lungs by vascular spread [7] and a route from the para-aortic lymph node and thoracic ducts to the mediastinum [8, 9]. The mechanism of mediastinal lymph node metastasis from the abdomen involves retrograde flow into the bronchomediastinal trunk from the thoracic duct [10]. We assumed that this was also the mechanism of the mediastinal lymph node metastasis in our case because the case involved gastric cancer in a site other than the cardia without lung metastasis and with a solitary superior mediastinal metastasis.

An FDG-PET scan has been reported as a useful diagnostic modality for advanced metastatic or recurrent gastric cancer, but not for detecting gastric cancer in signet ring cell and poorly differentiated adenocarcinoma, bone metastasis, peritonitis, or pleuritic carcinomatosis [11, 12]. Mediastinal lymph node metastases in advanced gastric cancer of the cardia without esophageal invasion occur occasionally, and those from sites other than the cardia are rare. A solitary superior mediastinal lymph node metastasis after distal gastrectomy is extremely rare; therefor, a PET scan is useful for the diagnosis of the lesion, which in this case, was not detected by CT. In contrast, this patient eventually developed multiple mediastinal metastasis, which is suggests that it is difficult for PET scan to detect small lesions (e.g. microscopic metastasis or peritoneal dissemination).

We found only two report of a solitary mediastinal metastasis in gastric cancer after gastrectomy in the Medline and Japana Centra Revuo Medicina databases [13, 14]. In first case, total gastrectomy with resection of the lower esophagus was performed for advanced gastric cancer of the cardia with slight invasion of the esophagus. Nine months later, a solitary middle mediastinal metastasis was detected and resected. The patient has been well and without recurrence for 4 years after resection of the metastatic tumor. In another case, distal gastrectomy was performed for advanced gastric cancer of the lower third of the stomach, five years later, a solitary thymic metastasis in the anterior mediastinum was detected and resected. The prognosis of the patient currently remains unclear.

Mediastinal lymph node metastasis from an adenocarcinoma in the gastroesophageal junction has been suggested as a prognostic factor [15]. An upper mediastinal lymph node metastasis in patients with gastric cancer often accompanies multiple metastases to other sites (e.g., Virchow^’^s lymph node); cases of a single mediastinal metastasis of gastric cancer after gastrectomy are rare. The two previously documented patients with superior mediastinal metastasis of gastric cancer that did not occur in the cardia received chemotherapy without surgery for superior mediastinal metastasis and were found to have multiple organ metastasis [5, 6]. A solitary recurrence is very rare in distant lymph node metastasis after gastrectomy of advanced gastric cancer. Therefore, resection of a distant lymph node metastasis is generally rare, but cases of radical dissection for a solitary axillary lymph node metastasis in gastric cancer have been also reported [16]. Furthermore, a patient with long-term disease-free survival after dissection of recurrent para-aortic lymph node metastases in gastric cancer has also been reported [17].

In our case, CT and PET scans did not clearly show any metastasis other than that in the solitary superior lymph node. The patient received oral S-1 as adjuvant chemotherapy but experienced recurrence and increased tumour markers. We predicted that tumor control with chemotherapy would be difficult; Hence, resection was recommended. The metastasized lymph node showed strong adhesion to the right brachiocephalic vein, but it was peripheral of the superior vena cava; therefore, it could be excised with the right brachiocephalic vein. We initially thought that the excision of the metastasized lymph node was curative because the tumor markers normalized postoperatively, however, the patient developed recurrence in the superior mediastinum and several right costa six months following reoperation.

In conclusion, we present a rare case of solitary metastasis to a superior mediastinal lymph node after distal gastrectomy for gastric cancer. A PET scan was useful for the diagnosis of mediastinal lymph node metastasis of gastric cancer. Metastasis of gastric cancer to a superior mediastinal lymph node implies systemic expansion of gastric cancer from sites other than the cardia; therefore, even if solitary, metastasis suggests a poor prognosis.

## Abbreviations

CA: Carbohydrate antigen
CEA: Carcinoembryonic antigen
CT: Computed tomography
FDG-PET: ^18^F- fluorodeoxyglucose positron emission tomography
